# Antibacterial and Anti-Biofilm Activity of Pyrones from a *Pseudomonas mosselii* Strain

**DOI:** 10.3390/antibiotics11111655

**Published:** 2022-11-18

**Authors:** Xueling Liu, Yali Wang, Diana A. Zaleta-Pinet, Robert P. Borris, Benjamin R. Clark

**Affiliations:** 1 Department of Pharmacy, The Affiliated Cancer Hospital of Zhengzhou University & Henan Cancer Hospital, Zhengzhou 450008, China; 2 School of Pharmaceutical Science and Technology, Health Sciences Platform, Tianjin University, Tianjin 300072, China; 3 College of Pharmacy, North China University of Science and Technology, Tangshan 063000, China

**Keywords:** biofilm, natural products, pseudopyronines, antibiotics, *Staphylococcus aureus*

## Abstract

The emergence of drug resistant microbes over recent decades represents one of the greatest threats to human health; the resilience of many of these organisms can be attributed to their ability to produce biofilms. Natural products have played a crucial role in drug discovery, with microbial natural products in particular proving a rich and diverse source of antimicrobial agents. During antimicrobial activity screening, the strain *Pseudomonas mosselii* P33 was found to inhibit the growth of multiple pathogens. Following chemical investigation of this strain, pseudopyronines A-C were isolated as the main active principles, with all three pseudopyronines showing outstanding activity against *Staphylococcus aureus*. The analogue pseudopyronine C, which has not been well-characterized previously, displayed sub-micromolar activity against *S. aureus*, *Staphylococcus epidermidis* and *Pseudomonas aeruginosa*. Moreover, the inhibitory abilities of the pseudopyronines against the biofilms of *S. aureus* were further studied. The results indicated all three pseudopyronines could directly reduce the growth of biofilm in both adhesion stage and maturation stage, displaying significant activity at micromolar concentrations.

## 1. Introduction

One of the current greatest threats to human health is that of drug resistant microbes, the increase in which can be attributed, in part, to the overuse of antibiotics [1]. In many cases, this trend is mediated by biofilm production in pathogens, which can increase the resistance of microbes to antibiotics and immune system responses. Biofilms are formed by the adhesion of a large number of bacteria to a contact surface, leading to the secretion of a mixture of polysaccharides and lipoproteins, enclosing the microbial colony and forming a membrane-like structure [2]. One notorious biofilm-forming pathogen is *Staphylococcus aureus,* a common hospital-acquired and food-borne bacterium [3]. It is widely distributed in air, water, dust, and the excreta of humans and animals. It can cause a variety of human and animal diseases [4], such as pseudomembranous colitis and sepsis [5], and is a serious threat to public health. Penicillin and methicillin have been widely used in the treatment of diseases caused by *S. aureus* [6], but increasing resistance has been observed, being largely related to the production of biofilms [7,8].

To overcome this situation, there is an urgent need for the development of novel agents that can effectively treat biofilm-producing *S. aureus*. One way is to develop new antimicrobial agents, while another approach is to combine existing antibiotic therapies with other compound that can inhibit biofilm production, in order to improve the efficacy of antimicrobials against severe and difficult infections. Historically, natural products have proven to be valuable sources of new antimicrobials or antimicrobial boosters [9,10,11]. Since the development of penicillin in the mid-20th century, natural products have been the source of the majority of antibiotics currently on the market [12]. More than 50% of clinical drugs were derived from or inspired by natural products between 1981 and 2019, and natural products still remain a valuable source of new drugs [13]. In particular, microbes are a rich source of antibiotics: all but three classes of antibiotics today have their roots in natural products of microbial origin, and microbes from a range of unusual environments continue to yield a wealth of novel chemical diversity [14,15,16,17,18].

Natural products containing an α-pyrone moiety can be produced by a variety of microorganisms and are widely distributed in nature; both naturally occurring pyrones and their synthetic derivatives have been found to exhibit a wide range of medicinal properties, including antibiotic and antifungal effects, which makes them an ideal target for drug development [19]. Two members of α-pyrone family, pseudopyronines A and B, were recently reported to possess moderate antibiotic properties. According to reports [20,21], pseudopyronines A and B inhibited the growth of multiple bacteria, including *Mycobacterium tuberculosis*, *Bacillus subtilis*, vancomycin-resistant *Enterococci*, methicillin-resistant *Staphylococcus aureus*, *Moraxella catarrhalis* and *Enterococcus faecium*. The mode of action appeared to be selective membrane disruption [22]. Among the pseudopyronines, pseudopyronine C has only been reported once previously [23], and the biological properties have not yet been evaluated. The effects of the pseudopyronines on microbial biofilms have also not been explored to date.

In this report, as a part of our ongoing studies into the chemistry of *Pseudomonas* species [24,25], we describe the isolation of antimicrobial compounds from an isolate of *Pseudomonas mosselii*. In our study, three pyrones, pseudopyronines A-C (**1**–**3**), along with three oxazoles, labradorins 1–2 and pimprinaphine (**4**–**6**), were isolated, and the antimicrobial and anti-biofilm properties of pseudopyronines were investigated in detail.

## 2. Results

### 2.1. Isolation and Structure Identification of Compounds ***1**–**6***

The bacterial strain P33 was isolated from a soil sample collected near Phang Nga Bay, Thailand. The strain was identified as *Pseudomonas mosselii* P33 based on sequence analysis of 16S rDNA. Well diffusion assays showed that strain P33 possessed significant activity against *Enterococcus hirae* (ATCC 8043), *Staphylococcus aureus* (ATCC 12600), and *Bacillus subtilis* (ATTCC 6633). In order to obtain the antibiotic principles, a large-scale culture of *Pseudomonas mosselii* (P33) was carried out, and the active components were isolated through a combination of liquid-liquid partition, column chromatography, and semi-preparative HPLC. The two major active components were identified by spectroscopic analysis as the pseudopyronines A (**1**) and B (**2**), along with the minor derivative pseudopyronine C (**3**). Three oxazoles: labradorin 1 (**4**), labradorin 2 (**5**), and pimprinaphine (**6**) were also isolated.

**Figure 1:**
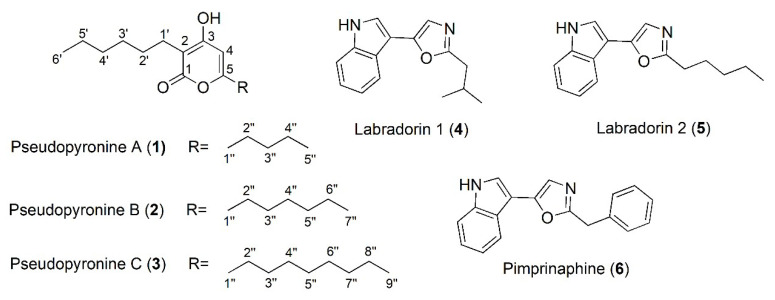
Structures of compounds **1**–**6**, isolated from *P. mosselii* P33.

Compounds **1**–**3** (Figure 1) were obtained as white, amorphous powders. They all shared similar UV-vis spectra with maximum absorptions at 293 nm, characteristic of an α-pyrone skeleton [26]. High-resolution MS spectra suggested molecular formulae of C_16_H_26_O_3_, C_18_H_30_O_3_, and C_20_H_34_O_3._ Comparison of spectroscopic data (Supplementary Information Figures S1–S9) confirmed the compounds to be the pseudopyronines A-C [23,26,27]. Compound **4** was isolated as a white, amorphous powder and identified as labradorin 1 based on NMR data and HRESIMS data (Supplementary Information Figures S10–S12). Compounds **5** and **6**, belonging to the same structural class, were identified as labradorin 2 and pimprinaphine by comparing the HRESIMS and NMR data (Supplementary Information Figures S13–S18) with those from previous reports [28,29].

### 2.2. Activities of Pseudopyronines A, B and C against Pathogens

According to prior reports [28], labradorins 1 and 2 were not active against multiple pathogens including *Staphylococcus aureus*, which was consistent with our results. Additionally, well diffusion assay showed that pimprinaphine was not active against any pathogenic bacteria used, while labradorin 1 showed only weak activity against *S. epidermidis*. Thus, further antimicrobial tests focused on the pseudopyronines were carried out. Prior studies have shown that pseudopyronines A and B possess good antibacterial activities against other microbial species [20,21,22], though the biological properties of pseudopyronine C, which has only been reported once previously [23], have not been evaluated previously. Thus, the pseudopyronines were assessed for antibacterial properties against an extended range of bacteria. The MICs of pseudopyronine A, B and C against seven ATCC pathogenic bacteria are shown in Table 1. The results indicated that all three compounds possessed antibacterial activities against *S. aureus*, *S. epidermidis* and *P. aeruginosa*, with pseudopyronines B and C in particular showing remarkable activity. Pseudopyronines A and B were also active against *E. hirae* and *B. subtilis*. Prior synthetic studies have shown that the difference in antimicrobial activities can be attributed to the differences in the lengths of the alkyl chains at C2 and C5, with pseudopyronines possessing 6–7 carbon alkyl chains possessing the highest activity against Gram-positive bacteria, such as *S. aureus* and *B. subtilis* [19].

### 2.3. Inhibition of Pseudopyronines on Biofilm Formation of S. aureus

#### 2.3.1. Activity of Pseudopyronines against Planktonic *S. aureus*

**Figure 2:**
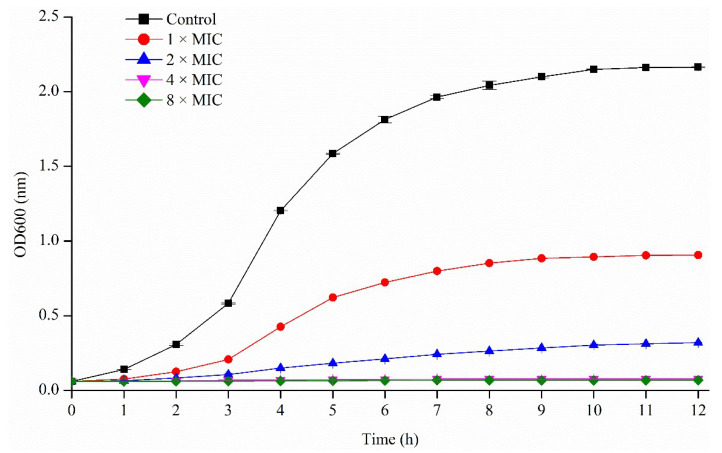
Growth curve of *S. aureus* with different concentrations of pseudopyronine B (1 × to 8 × MIC). The control group represents only *S. aureus* in the medium, without a test compound.

All three pseudopyronines A, B and C showed significant antibacterial activity against *S. aureus* with MICs (µg/mL) of 6.25, 0.156 and 0.39, respectively (Table 1). *S. aureus* is a common and worldwide pathogenic bacterium and threat to human health [3,4,5], Thus, the strong growth inhibition of *S. aureus* was of particular interest. To further investigate the antimicrobial properties of pseudopyronine B, the growth of *S. aureus* (Figure 2) was measured in the presence of different concentrations of pseudopyronine B. From the growth curve, significant growth inhibition of *S. aureus* could be seen at 1 × MIC in comparison to the control group, with almost complete growth inhibition of *S. aureus* at 4 × MIC.

#### 2.3.2. Inhibition of Pseudopyronines on *S. aureus* Biofilm Formation

**Figure 3:**
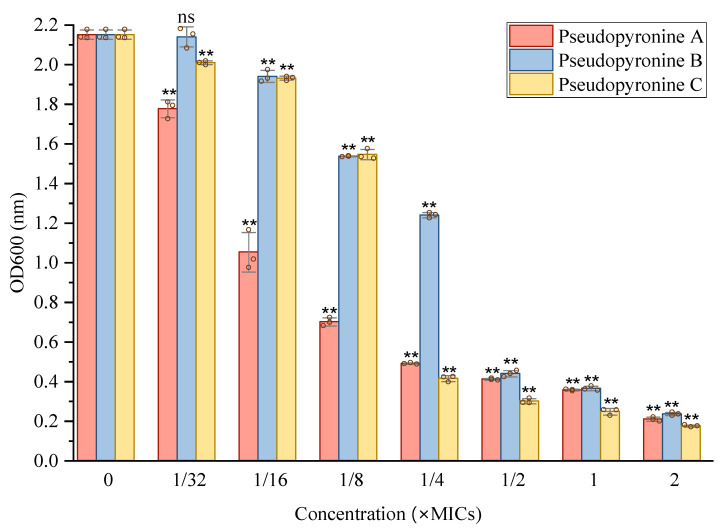
Effects of sub-MIC concentrations of pseudopyronine A, B and C on biofilm formation of *S. aureus*, as determined by crystal violet assay at different sub-MIC concentrations (1/32 × to 2 × MIC) Data are presented as mean ± SD, *n* = 3, ** *p* < 0.01, ^ns^ *p* ˃ 0.05 (not statistically significant), compared to the control group. Individual data points are shown as hollow circles, for reference.

*S. aureus* is known to produce biofilms, which can render it more difficult to eliminate compared with planktonic forms [6,7]. Thus, the anti-biofilm formation properties of the pseudopyronines were assessed. An overnight culture of *S. aureus* (ATCC 12600) was incubated together with various concentrations of pseudopyronines A-C. The inhibition of biofilm formation was determined by the crystal violet (CV) assay, with sub-MIC concentrations of all three pseudopyronines showing anti-attachment effects on *S. aureus* biofilms in comparison to the control group. According to the results (Figure 3), all of the pseudopyronines showed 80–90% inhibition at concentrations above 1/2 × MIC. Decreasing concentrations of the test samples led to a corresponding decrease in biofilm inhibition. These results showed that all three pseudopyronines can greatly inhibit biofilm formation of *S. aureus* at the initial adhesion stage.

#### 2.3.3. Reduction of Mature Biofilms of *S. aureus* by Pseudopyronines A-C

**Figure 4:**
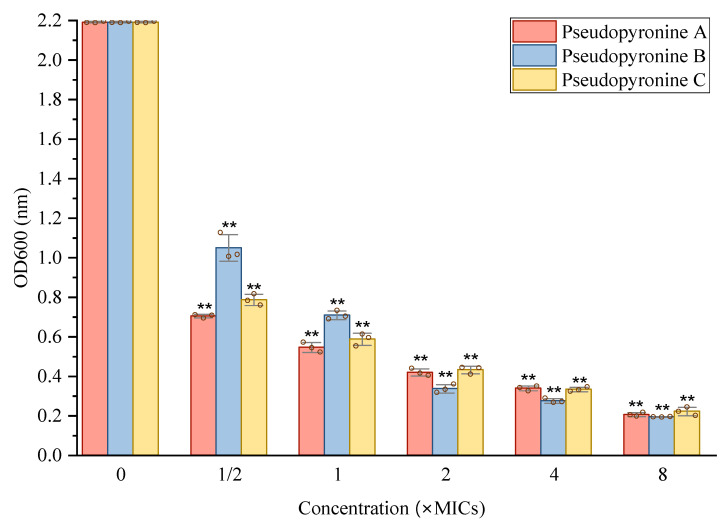
Reduction effects of sub-MIC concentrations of pseudopyronine A, B and C on mature biofilm of *S. aureus*. Data are presented as mean ± SD, *n* = 3, ** *p* < 0.01, compared to the control group.

**Figure 5:**
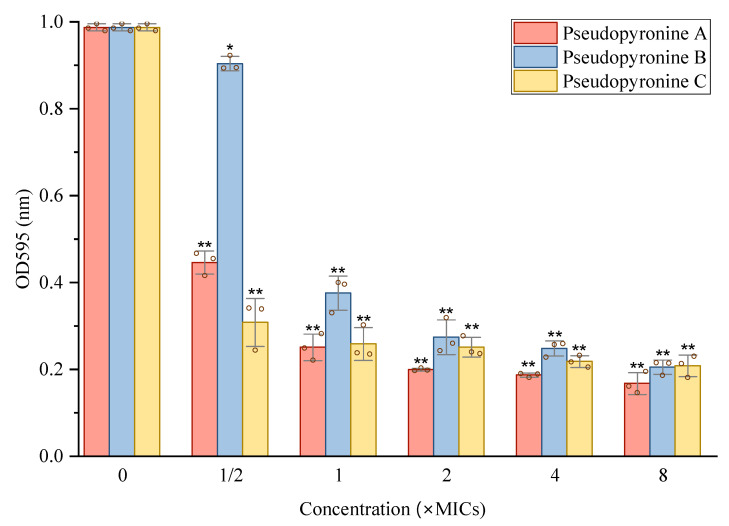
Reduction effects of sub-MIC concentrations of pseudopyronine A, B and C on metabolic activity of *S. aureus* evaluated by the MTT assay (1/2 × to 8 × MIC). Data are presented as mean ± SD, *n* = 3, * *p* < 0.05, ** *p* < 0.01, compared to the control group.

With the activity of the pseudopyronines against growing biofilms established, their activity against mature *S. aureus* biofilms was measured by two methods (Figure 4 and Figure 5). All three pseudopyronines could significantly reduce the mature biofilm of *S. aureus* at sub-MIC concentrations, based on the crystal violet staining assay, which showed reduction of the mature biofilm. Inhibition values of 80–90% were observed at concentrations above 2 × MIC (Figure 4). In order to further study the effects of pseudopyronines on *S. aureus* biofilms, the metabolic activity of any viable bacteria in the biofilm was assessed by MTT assay at the same concentrations (Figure 5). The results indicated that all three pseudopyronines could produce a 75–90% reduction of metabolic activity at concentrations above 2 × MIC, with biofilms grown in the presence of pseudopyronines having significantly lower metabolic activity compared to the untreated control, which was consistent with the previous crystal violet experimental results. Both the crystal violet staining assay and MTT assay confirmed that pseudopyronines could directly kill bacteria in the mature biofilm.

**Figure 6:**
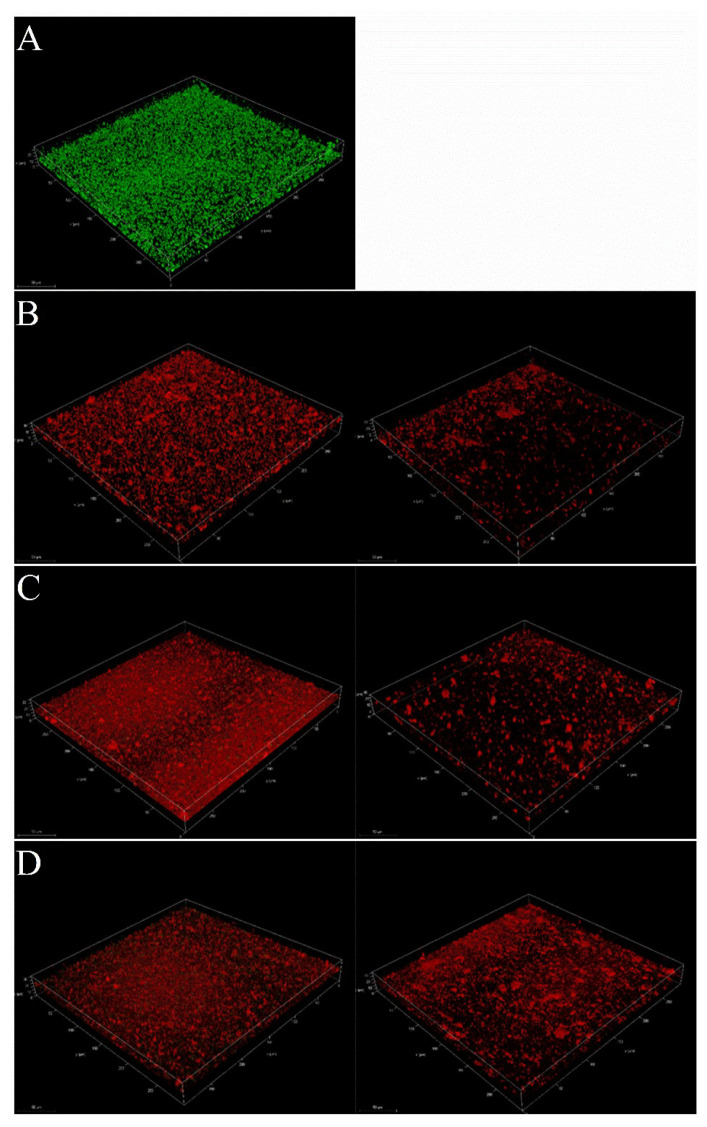
Confocal Laser Scanning Microscopic 3D images of *Staphylococcus aureus* biofilms. (**A**) negative control. (**B**) sub-MIC concentrations of pseudopyronine A. Left-2 × MIC; Right-8 × MIC. (**C**) sub-MIC concentrations of pseudopyronine B. Left-2 × MIC; Right-8 × MIC. (**D**) sub-MIC concentrations of pseudopyronine C. Left-2 × MIC; Right-8 × MIC. Green and red fluorescence represent live and dead cells, respectively.

Further investigations were carried out using confocal microscopy. 3D biofilm images of mature biofilms, produced with different sub-MIC concentrations of the three pseudopyronines, were obtained using a confocal laser scanning microscope (Figure 6). After 24 h incubation, the pseudopyronines were added to the pre-formed biofilm and incubated for another 24 h, then images were obtained. In the absence of pseudopyronines, the biofilm (in green) was distributed uniformly and possessed high density, covering the glass surface entirely (Figure 6A). Following treatment with pseudopyronines, red fluorescence (dead cells) largely increased and green fluorescence (live cells) greatly decreased (Figure 6B–D), which suggested that the pseudopyronines penetrated the biofilm and further disrupted the pre-formed biofilms. Moreover, biofilms were visibly diffuse, with decreased thickness (Figure 6B–D). All three of the pseudopyronines could still greatly disrupt the construction of the pre-formed biofilm, with pseudopyronines A and B having the highest activity. Taken together, these results suggested that three pseudopyronines can penetrate the *S. aureus* biofilm and alter biofilm architecture.

## 3. Discussion

Biofilm formation is a crucial factor in human infections, especially owing to its ability to affect drug resistance in pathogens [7,8]. Bacteria which produce biofilms are often more difficult to eliminate, compared with their planktonic forms. Biofilms are composed of a network of microbial communities surrounded by an extracellular matrix, the formation of which is mediated by a cell–cell signaling system: quorum sensing (QS). The formation of biofilms can be divided into two major processes, including the initial adherence, followed by maturation into a complex three-dimensional architecture [30]. One of the avenues available in addressing the problem of biofilms is to prevent biofilm adhesion [31], while other studies focus on killing the bacteria in mature biofilms.

*S. aureus* is one of the most common gram-positive opportunistic pathogens, which can easily produce a strong biofilm associated with long-term infections and remains a difficult challenge to handle in clinical settings. Singh et al. indicated that the antibacterial mechanism of pseudopyronines A and B against *B. subtilis* was on the basis of selective membrane disruption and inhibition of fatty-acid synthase (FAS) II (FabG/FabI/InhA) [22]. The present work indicates that pseudopyronines A-C have strong antibacterial activity against *S. aureus* with MICs (µg/mL) of 6.25, 0.156 and 0.39, respectively. Previous studies conducted by Singh showed that MICs of pseudopyronines A and B against *S. aureus* were 2–16 µg/mL, the difference here may owe to the different microbial strain used [22]. The anti-biofilm effects of pseudopyronines have not been studied previously; this study represents the first report of the anti-biofilm properties of these compounds. The crystal violet assay indicated that pseudopyronines A, B and C were active against biofilm formation at a concentration from 0.3–20 µM (Figure 3), in particular pseudopyronines B (0.3 µM) and C (0.6 µM), which could inhibit over 80% adhesion in *S. aureus* and significantly reduce biofilm formation. This activity is significant, even when compared with other reference antibiofilm agents such as baicalein, nifuroxazide, tannic acid, and the most active, 5-aryl-2-aminoimidazole derivatives, which can inhibit biofilm formation of *S. aureus* by 50% at a concentration of 2.8 µM [32].

The crystal violet staining assay, along with the MTT assay, showed that all three pseudopyronines could directly eliminate the mature biofilm and lower biofilm metabolic activity as compared to control groups. Moreover, the reduction of mature biofilms by the pseudopyronines was further confirmed by confocal laser scanning microscopy analysis, where the 3D images showed that the structure of the biofilm was markedly disrupted with treatment with pseudopyronines A-C (Figure 6). These results showed that pseudopyronines B and C could still present strong reduction activity in mature biofilm, even at low concentrations. Compared with some other antibiofilm agents such as the polyphenol carnosol, which possesses strong antibiofilm activity during biofilm formation, but low activity (1.54 mM) in the mature stage, these results are significant [11].

## 4. Materials and Methods

### 4.1. General

Optical density was recorded on a UV spectrophotometer (UV1800, Shimadzu, Japan). Ultra-pure water was obtained from the Direct-Q^®^ 5UV instrument (Merck Millipore, Billerica, MA, USA). High-resolution electrospray ionization mass spectra (HR-ESI-MS) were measured using a Q Exactive HF Orbitrap LC-MS (Thermo Fisher Scientific, Waltham, MA, USA). NMR spectra were recorded on an Avance III 600 MHz spectrometer (Bruker BioSpin, Billerica, MA, USA) using TMS or the residual solvents as internal standard. Analytical and semipreparative HPLC separations were performed on an Agilent 1260 series HPLC system (G1311B quaternary pump, G1329B autosampler, G1316A column compartment and G1315D photodiode array detector, Agilent Technologies; Santa Clara, CA, USA), using a Pursuit XRs C18 column (5 µm, 4.6 × 150 mm, Agilent Technologies, USA). Semipreparative HPLC separation was carried out on a Shimadzu LC-20AR series HPLC instrument equipped with a UV detector and a reversed-phase C18 column (Pursuit XRs-C18, 10 µm, 21.2 × 250 mm). All solvents used were of HPLC grade (Concord Technologies, Tianjin, China). Thin-layer chromatography (TLC) was carried out on silica gel GF_254_ plates (Haiyang Chemicals Corp., Qingdao, China). Sterile cell grade 96-well microtiter plates (Costar 3599) were obtained from Corning (New York, NY, USA). All antibiotics were purchased from Sigma-Aldrich Chemical Co. (St. Louis, MO, USA). Nutrient agar was purchased from AOBOX (Beijing, China). Brain heart infusion broth and potato dextrose broth were purchased from Solarbio (Beijing, China). YM medium was purchased from Hopebio (Qingdao, China), and agar was purchased from Guangfu-chem (Tianjin, China). Incubation and biological operations involving microorganisms were performed in constant temperature incubators and super clean bench (Shanghai Zhicheng Analysis Instrument Manufacturing Co., Ltd., Shanghai, China), respectively. High temperature sterilization was performed with an autoclave (Zhiwei Instrument Co., Ltd., Xiamen, China). Optical density for microbial strains was read on a microplate reader (Multiskan GO, Thermo Scientific, Waltham, MA, USA). Sterile Phosphate Buffered Saline and cell grade DMSO were purchased from Solarbio life sciences (Beijing, China).

### 4.2. Bacterial Strains and Materials

Strains of *Staphylococcus aureus* (ATCC 12600), *Enterococcus hirae* (ATCC 8043), *Streptococcus mutans* (ATCC 25175), *Moraxella catarrhalis* (ATCC 25238), *Staphylococcus epidermidis* (ATCC 14990), *Pseudomonas aeruginosa* (ATCC 15692), *Bacillus subtilis* (ATCC 6633) were purchased from American Type Culture Collection Center.

Pseudopyronines A, B and C were isolated from a bacterial strain *Pseudomonas mosselii* P33, which was isolated from a soil sample collected near Phang Nga Bay, Thailand in late December 2016. The strain was identified based on sequence analysis of 16S rDNA, the sequence has already been submitted to the NCBI as GenBank accession number SUB9332606 SequenceP33ID MW786754. Purified microbial strain P33 was used for DNA extractions. Isolation and amplification of DNA, and sequence analysis were carried out according to previous reports. Firstly, extraction of DNA was conducted as follows. Microbes were first cultured on an agar plate until the ideal colony size (2–3 mm) was formed. A sterilized Eppendorf tube containing 567 μL TE buffer was prepared, then the 3 mm colony was transferred to the tube using an inoculation loop. The solution was mixed by repeating pipetting. Then, 30 μL 10% SDS and 3 μL Proteinase K (20 mg/mL) were added to the solution and incubated at 37 °C for 1 h. After incubation, 100 μL NaCl (5 M) was added, then 80 μL CTAB/NaCl solution (0.7 M NaCl, 10% CTAB) was added, the mixture was incubated at 65 °C for 10 min. After incubation, to remove protein, 780 μL phenol: chloroform: isoamyl alcohol (25:24:1) was added and mixed, then the mixture was centrifuged for 5 min at 14,000 rpm. Finally, the aqueous solution was transferred to a new tube. This step was repeated using chloroform: isoamyl alcohol (24:1). Afterwards, 0.6 volume of isopropanol was added and mixed gently until DNA precipitated. The mixture was centrifuged again to remove isopropanol from the DNA, 1 mL ethanol (70%) was added to wash the salt away. After centrifugation, ethanol was discarded, and the purified DNA was dried on the benchtop at room temperature. After drying, the DNA was re-suspended in DNase/RNase-free water and was kept at −20 °C. Secondly, Polymerase chain reaction (PCR) experiment was applied, DNA concentration was measured with an Infinite^®^ 200 PRO plate reader (TECAN, Männedorf, Switzerland), DNase/RNase- water was used as control. The 16S rDNA genes were amplified using PrimeSTAR^®^ Hs DNA Polymerase (Takara, Dalian, China). The total reaction volume was 50 μL. The mixture contained 10 μL 5× Primer Buffer, 4 μL dNTP Mixture (2.5 mM), 1 μL of each 1:50 diluted primer 27F (5’-AGAGTTTGATC CTGGCTCAG-3’) and 1541R (5’-AAGGAGGTGATCCAG CCG CA-3’), 0.5 μL Hs DNA Polymerase, 200 ng DNA template and RNA-free water. PCR amplification was performed in Mastercycler Nexus gradient (Eppendorf, Germany). Amplification of DNA was carried out under the following conditions: 30 cycles of denaturation at 95 °C for 10 s, annealing at 50 °C for 15 s and extension at 72 °C for 90 s. Then, electrophoresis and sequence analysis were carried out, and the quality of the PCR products was assessed by electrophoresis. Gel was prepared from 0.5× TBE buffer with 1% agarose, the solution was heated until the agarose was dissolved. Ethidium bromide (0.5 μg/mL) was added when the molten gel has cooled, then it was poured into a mold with appropriate comb, later 0.5× TBE buffer was added to cover the gel to a depth of 1 mm.

The samples of DNA were mixed with 6× gel-loading buffer. The electrophoresis was set at 110 V, 80 mA for 90 min at room temperature. Ideally, DNA fragments within the appropriate size (1200–1600 bp) were observed under the UV lights at 280 nm.

The PCR products were sequenced by TsingKe Biological Technology (Beijing). Finally, the 16S rDNA gene sequences were analyzed and compared to sequences at National Center for Biotechnology Information (NCBI). The 16S rDNA gene similarity values were calculated by comparison of the sequences with the alignments, the phylogenetic trees were constructed at NCBI to determine the genus and species of the bacteria. 16S rDNA sequence of P33 and phylogenetic trees could be found in the supporting information (Supplementary Information Figure S20).

### 4.3. Large-Scale Extraction and Isolation

A large-scale culture (20 L) of *Pseudomonas mosselii* P33 was carried out in PDB medium at 30 °C, shaking at 200 rpm for 7 days. The medium was extracted with CH_2_Cl_2_ (10 L) and the biomass with methanol (5 L), followed by filtration. The CH_2_Cl_2_ and methanol extracts were combined and evaporated to dryness *in vacuo* to afford a combined residue (4.8 g). The organic extract was then dispersed in MeOH:H_2_O (9:1, 100 mL) and defatted with *n*-hexane (3 × 200 mL). The hydroalcoholic phase was dried again under vacuum, dispersed in water and successively extracted with dichloromethane (CH_2_Cl_2_) and *n*-BuOH (each 3 × 200 mL) to afford *n*-hexane (2.2 g), CH_2_Cl_2_ (2.3 g), *n*-BuOH (1.0 g) and aqueous (1.2 g) fractions.

The *n*-hexane fraction (2.2 g), together with CH_2_Cl_2_ (2.3 g) fraction was separated on silica gel (200 g) open column chromatography by eluting with a CH_2_Cl_2_-EtOAc step gradient (CH_2_Cl_2_→100% EtOAc, each 250 mL) to give 8 sub-fractions. Fr. 4 (523.0 mg), together with Fr. 5 (486.2 mg) were subjected to semi-preparative HPLC (Pursuit XRs-C18, 10 µm, 21.2 × 250 mm) using isocratic elution with 65% ACN-H_2_O at 10 mL/min for the first 125 min, followed by washing with 100% ACN for 25 min, which then afforded pure pseudopyronine A (**1**) (12.6 mg) and pseudopyronine B (**2**) (72.5 mg).

Fr. 6 (232.2 mg) was separated on the same semi-preparative HPLC column with the isocratic elution of 82 % MeOH-H_2_O at 10 mL/min for 70 min, followed by washing with 100% MeOH for another 30 min, which afforded pure pseudopyronine C (**3**) (8.6 mg).

Fr. 3 (300.5 mg) was separated on the same semi-preparative HPLC column with an isocratic elution of 70% MeOH-H_2_O at 5 mL/min for 85 min, followed by washing with 100% MeOH for another 30 min, which afforded pure labradorin 1 (**4**) (15.3 mg), labradorin 2 (**5**) (4.2 mg) and pimprinaphine (**6**) (8.2 mg).

### 4.4. Biofilm Inhibition against Staphylococcus aureus

#### 4.4.1. Determination of Minimum Inhibitory Concentration (MIC)

The MICs of pseudopyronines A, B and C and the antibiotics penicillin, ampicillin, vancomycin, cephalexin and ciprofloxacin were determined against seven ATCC strains using the reference protocol of the Clinical and Laboratory Standards Institute (2013) broth method [33]. Briefly, overnight cultures were diluted to 10^8^ CFU/mL in fresh TSB or BHI media. Serial two-fold dilutions were prepared in TSB or BHI media in sterile 96-well microtiter plates at 37 °C for 24 h, then their OD values were measured at 600 nm. The MIC was defined as the lowest drug concentration at which no visible bacterial growth was observed.

#### 4.4.2. Growth Curve for *S. aureus*

An overnight culture of *S. aureus* was used to inoculate a fresh TSB culture in five 250 mL Erlenmeyer flasks (50 mL, OD_600_ = 0.06) with different concentrations of pseudopyronine B (1 × to 8 × MIC). The broth medium cultures were incubated at 37 °C in the shaker. The cell density was measured at 600 nm every 0.5 h up to 12 h using a spectrophotometer (UV1800, Shimadzu, Japan). Each experiment was performed with triplicate samples.

#### 4.4.3. Inhibition of Cell Attachment

The crystal violet assay was applied to measure the whole biofilm biomass in flat-bottom 96-well polystyrene microtiter plates, based on a previous method with slight modification [34,35]. Overnight cultures of *S. aureus* were diluted into fresh TSB medium and adjusted to a final concentration of 1 × 10^8^ CFU/mL. For the screening of test samples, wells of 96-well microtiter plates were filled with 200 µL of the diluted cultures with test samples at sub-MIC concentrations (1/32 × to 2 × MIC), or DMSO, in triplicate. The plates were incubated at 37 °C for 48 h. After incubation, the medium was gently removed and the plates were washed twice with sterile phosphate-buffered saline (PBS). Biofilms attached to test microtiter wells were then stained with 200 µL of 0.1% (*w*/*v*) crystal violet solution (Solarbio, Beijing, China) and allowed to stand at room temperature for 30 min. The excess crystal violet solution was removed, and well was washed three times with PBS. The attached crystal violet was dissolved by adding 150 µL 95% ethanol. Absorbances were measured at a wavelength of 595 nm by microplate reader. Experiments were performed with triplicate samples.

#### 4.4.4. Reduction of Biofilm Growth

The inhibition of biofilm formation by pseudopyronines A-C was assessed using the crystal violet assay with minor modifications [36,37]. Briefly, overnight cultures grown in TSB were diluted to 10^8^ CFU/mL in TSB. To activate the formation of biofilm, a 200 µL aliquot culture medium was transferred into a flat-bottom 96-well polystyrene microtiter plate. After incubation at 37 °C for 24 h, the medium was gently removed, then each well was rinsed three times with sterile cell grade PBS. A total of 200 µL of pseudopyronines A, B and C (1/2 × to 8 × MIC) were added to the wells, followed by incubation at 37 °C for 24 h. After the incubation, the plates were washed with PBS to remove non-adherent cells. Next, the wells were stained with 200 µL of 0.1% (*w*/*v*) crystal violet for 30 min at room temperature. In order to remove the excess stain, the plates were further washed three times with PBS. The mean absorbance (OD_595_) of the crystal violet-stained biofilm cells was obtained, and the percent inhibition of pseudopyronines A, B and C were determined by the following formula: [(OD _growth control_ − OD _sample_)/OD _growth control_] × 100.

#### 4.4.5. Metabolic Activities of Pre-Formed Biofilm

The metabolic activity of biofilms formed in the 96-well plate was assessed by MTT [3-(4,5-dimethylthiazol-2-yl)-2,5-diphenyltetrazolium bromide] assay with minor modifications [38,39]. The biofilm was formed over 24 h and then treated with pseudopyronines A-C (24 h at 37 °C) in a 96-well microtiter plate. After incubation, planktonic bacteria were discarded carefully and the biofilms in the wells were washed with sterile PBS. 200 µL of 0.5 mg/mL MTT dissolved in sterile PBS was added to each test well and incubated at 37 °C in the dark. After 4 h, the liquor in the wells was gently removed, followed by adding DMSO (150 µL) to dissolve the formazan crystals. OD_595nm_ was determined using a microplate reader. Experiments were performed in triplicate.

#### 4.4.6. Confocal Laser Scanning Microscopy (CLSM) Analysis

Overnight bacterial cultures at (1 mL, OD_600 nm_ = 0.1 ± 0.02) were transferred to microscope slides to initiate growth of biofilm, followed by incubation at 37 °C for 24 h. Slides were then washed twice with sterile PBS. Then, pseudopyronines A-C were prepared in a volume of 1 mL bacterial cultures at 2 × MIC and 8 × MIC concentrations, then transferred to the microscope slides, followed by another incubation at 37 °C for 24 h. Slides were again washed as described above and stained for 30 min in the dark with a LIVE/DEAD BacLight bacterial viability kit (Molecular Probes, Eugene, OR, United States). Biofilm Image analysis was performed on a confocal laser scanning microscope (Leica TCS SP8, Leica microsystems, Wetzlar, Germany) with excitation at 490 nm and emission at 515 nm and 617 nm for Syto 9 (live cells in green color) and propidium iodide (damaged and dead cells in red color) staining, respectively. All biofilms were obtained as 3D images at ×100 magnification.

### 4.5. Statistical Analysis

Statistical analysis was performed using a one-way ANOVA following Tukey’s test on Origin Pro 8.5 software, performed according to controls in each dataset. Data are presented as mean ± standard deviation (SD). The significance level was set to *p* < 0.05 and *p* < 0.01.

## 5. Conclusions

In conclusion, the six isolated compounds were reported for the first time from *Pseudomonas mosselii*, though they have been described from other *Pseudomonas* species [28,29]. We also describe previously unreported antimicrobial properties for the pseudopyronines, with all three possessing outstanding antimicrobial activity against a range of different pathogens. In particular, pseudopyronine C has only been reported once previously, and no biological properties were described [23]. The significant anti-biofilm activity detected for the pseudopyronines suggests they could have a potential application against *S. aureus* biofilms at both the adhesion stage and the maturation stage. As biofilms are regulated by multiple biological systems, further studies can be applied to understand the molecular mechanism of pseudopyronines on *S. aureus* biofilm inhibition, and effects on clinically relevant pathogenic bacteria. Considering that pseudopyronines showed both activity against multiple bacterial species and significant ability to affect biofilm formation, these compounds could be modified to produce new compounds with better antimicrobial activity, which could provide alternative strategies for the discovery of new antibiotics in the future.

## Figures and Tables

**Table 1:** MICs of pseudopyronines A, B and C against multiple pathogenic bacteria.

Compound	MIC (µg/mL)						
	*S. aureus*	*S. epidermidis*	*P. aeruginosa*	*S. mutans*	*M. catarrhalis*	*E. hirae*	*B. subtilis*
Pseudopyronine A	6.25	12.5	12.5	--	--	12.5	25
Pseudopyronine B	0.156	1.0	1.56	1.56	1.56	3.125	12.5
Pseudopyronine C	0.39	1.56	0.78	--	--	--	--
Positive control	0.01 ^a^	0.02 ^a^	0.0975 ^b^	2.5 ^c^	6.25 ^d^	0.78 ^c^	2.5 ^e^

-—No activity; ^a^—Penicillin, ^b^—Ciprofloxacin, ^c^—Ampicillin, ^d^—Cephalexin, ^e^—Vancomycin.

## Data Availability

The data presented in this study are available on request from the corresponding author.

## Supplementary Materials

The following supporting information can be downloaded at: https://www.mdpi.com/article/10.3390/antibiotics11111655/s1. Structure elucidation of compounds **1**–**6**. Table S1: ^1^H (600 MHz) and ^13^C NMR (150 MHz) data for pseudopyronine A, B and C with *J* values (in Hertz) in parentheses. Figure S1: HRESIMS spectrum of known compound **1**. Figure S2: ^1^H-NMR spectrum of known compound **1**. Figure S3: ^13^C-NMR spectrum of known compound **1**. Figure S4: HRESIMS spectrum of known compound **2**. Figure S5: ^1^H-NMR spectrum of known compound **2**. Figure S6: ^13^C-NMR spectrum of known compound **2**. Figure S7: HRESIMS spectrum of known compound **3**. Figure S8: ^1^H-NMR spectrum of known compound **3**. Figure S9: ^13^C-NMR spectrum of known compound **3**. Figure S10: HRESIMS spectrum of known compound **4**. Figure S11: ^1^H-NMR spectrum of known compound **4**. Figure S12: ^13^C-NMR spectrum of known compound **4**. Figure S13: HRESIMS spectrum of known compound **5**. Figure S14: ^1^H-NMR spectrum of known compound **5**. Figure S15: ^13^C-NMR spectrum of known compound **5**. Figure S16: HRESIMS spectrum of known compound **6**. Figure S17: ^1^H-NMR spectrum of known compound **6**. Figure S18: ^13^C-NMR spectrum of known compound **6**. Figure S19: Antimicrobial assay of methanolic extract of *P. mosselii* P33. Figure S20: Phylogenetic tree of *P. mosselii* P33.
