# Supplementary material for: Antibacterial and Anti-Biofilm Activity of Pyrones from a Pseudomonas mosselii Strain
Source: Antibiotics (Basel). 2022 Nov 18;11(11):1655. doi: 10.3390/antibiotics11111655 (PMC9686599; doi:10.3390/antibiotics11111655)
Supplement: Supplementary file 1 [file antibiotics-11-01655-s001.zip › antibiotics-2009023-supplementary.pdf]

Supporting Information

Antibacterial and anti-biofilm activity of pyrones from a *Pseudomonas mosselii* strain

Xueling Liu*, Yali Wang, Diana A. Zaleta-Pinet, Robert P. Borris, Benjamin R. Clark*

Contents

Structure elucidation of compounds **1**-**6**. 2

Figure S1 HRESIMS spectrum of known compound **1** 4

Figure S2 ^{1}H-NMR spectrum of known compound **1** (Methanol-d_4, 600 MHz) 4

Figure S3 ^{13}C-NMR spectrum of known compound **1** (Methanol-d_4, 150 MHz) 5

Figure S4 HRESIMS spectrum of known compound **2** 5

Figure S5 ^{1}H-NMR spectrum of known compound **2** ($CDCl_3$, 600 MHz) 6

Figure S6 ^{13}C-NMR spectrum of known compound **2** ($CDCl_3$, 150 MHz) 6

Figure S7 HRESIMS spectrum of known compound **3** 7

Figure S8 ^{1}H-NMR spectrum of known compound **3** (Methanol-d_4, 600 MHz) 7

Figure S9 ^{13}C-NMR spectrum of known compound **3** (Methanol-d_4, 150 MHz) 8

Figure S10 HRESIMS spectrum of known compound **4** 8

Figure S11 ^{1}H-NMR spectrum of known compound **4** (Methanol-d_4, 600 MHz) 9

Figure S12 ^{13}C-NMR spectrum of known compound **4** (Methanol-d_4, 150 MHz) 9

Figure S13 HRESIMS spectrum of known compound **5** 10

Figure S14 ^{1}H-NMR spectrum of known compound **5** (Methanol-d_4, 600 MHz) 10

Figure S15 ^{13}C-NMR spectrum of known compound **5** (Methanol-d_4, 150 MHz) 11

Figure S16 HRESIMS spectrum of known compound **6** 11

Figure S17 ^{1}H-NMR spectrum of known compound **6** ($CDCl_3$, 600 MHz) 12

Figure S18 ^{13}C-NMR spectrum of known compound **6** ($CDCl_3$, 150 MHz) 12

Figure S19 Antimicrobial assay of methanolic extract of *P. mosselii* P33 13

Figure S20 Phylogenetic tree of *P. mosselii* P33 14

Structure elucidation of compounds **1**-**6**.

Table S1 ^{1}H (600 MHz) and ^{13}C NMR (150 MHz) data for pseudopyronine A, B and C with *J* values (in Hertz) in parentheses.

	Pseudopyronine A[a]		Pseudopyronine B[b]		Pseudopyronine C[a]	
Position	H	C	H	C	H	C
1		169.5		167.8		169.0
2		103.6		103.4		103.8
3		169.1		166.3		168.9
4	5.93 (1H, s)	101.1	6.12 (1H, s)	100.5	5.95 (1H, s)	101.7
5		164.9		163.7		164.9
1'	2.36 (2H, t, 7.5)	23.9	2.43 (2H, m)	23.1	2.36 (2H, t, 7.8)	23.9
2'	1.44 (2H, m)	29.1	1.50 (2H, m)	28.1	1.44 (2H, m)	29.1
3'	1.32 (2H, o)	30.3	1.30 (2H, o)	29.4	1.33 (2H, o)	30.2
4'	1.31 (2H, o)	33.0	1.30 (2H, o)	31.7	1.31 (2H, o)	32.9
5'	1.31 (2H, o)	23.7	1.30 (2H, o)	22.6	1.31 (2H, o)	23.7
6'	0.89 (3H, t, 6.9)	14.4	0.87 (3H, o)	14.1	0.89 (3H, o)	14.4
1''	2.44 (2H, t, 7.5)	34.3	2.44 (2H, m)	33.5	2.44 (2H, t, 7.5)	34.3
2''	1.64 (2H, m)	27.6	1.62 (2H, m)	26.8	1.63 (2H, m)	27.9
3''	1.34 (2H, o)	32.2	1.30 (2H, o)	29.0	1.35 (2H, o)	30.0
4''	1.35 (2H, o)	23.4	1.30 (2H, o)	31.8	1.31 (2H, o)	33.0
5''	0.91 (3H, t, 6.8)	14.2	1.30 (2H, o)	22.7	1.31 (2H, o)	30.5
6''			0.87 (3H, o)	14.1	1.31 (2H, o)	30.4
7''					1.31 (2H, o)	32.9
8''					1.31 (2H, o)	23.7
9''					0.89 (3H, o)	14.4

[a]- in methanol-d_4; [b]- in $CDCl_3$; o-overlapped

Compounds **1**, **2** and **3** were obtained as white, amorphous powders. They all shared similar UV-vis spectra with maximum absorptions at 293 nm, characteristic of an α-pyrone

skeleton[21]. Compound **1** was isolated as a white solid. The molecular formula was determined to be $C_{16}H_{26}O_3$ based on the observation of an $[M + H]^+$ quasi-molecular ion at *m*/*z* 267.1958 (calcd. for $C_{16}H_{27}O_3$, 267.1955) in the HR-ESI-orbitrap mass spectrum, the NMR data agreed with previous report. Therefore, compound **1** was identified as pseudopyronine A[21]. Compound **2** and **3** possessed similar NMR spectra to compound **1**, differing only in the length of the alkyl chains; they were identified as pseudopyronines B and C, respectively[21–23].

The molecular formula for compound **2** was determined to be $C_{18}H_{30}O_3$ based on the observation of an $[M + H]^+$ quasi-molecular ion at *m*/*z* 295.2270 (calcd. for $C_{18}H_{31}O_3$, 295.2268) in the HR-ESI-orbitrap mass spectrum. ^{1}H NMR data revealed the presence of two linear alkyl chains, and a single aromatic proton. In combination with the ^{13}C-NMR and DEPT spectra, compound **2** included two methyl groups, eleven methylene groups and one methine. In the HMBC spectrum, correlations from H-1' (δ 2.43) to C-1 (δ 167.8), C-3 (δ 166.3) and correlations from H-1'' (δ 2.44) to C-4 (δ 100.5) confirmed that two alkyl chains were located at C-2 and C-5 of the α-pyrone moiety, respectively. All of the individual protons and carbons (Table S1) were assigned by a combined analysis of 1D and 2D NMR analysis; this was sufficient to identify the compound as pseudopyronine B[23].

Compound **3** was isolated as a white amorphous powder. The molecular formula was determined as $C_{20}H_{34}O_3$ based on the observation of an $[M + H]^+$ quasi-molecular ion at *m*/*z* 323.2576 (calcd. for $C_{20}H_{35}O_3$, 323.2581) in the HRESIMS spectrum. All of the individual protons and carbons (Table S1) were assigned by a combined analysis of 1D and 2D NMR. According to the spectral features and the same UV absorption suggested that compound **3** possessed the same α-pyrone moiety with two fatty acid chain substituents. Comparing the molecular weight of compound **2** and **3**, suggested that compound **3** contained two more methylene groups. In the HMBC spectrum, correlations from H-1' (δ 2.36) to C-1 (δ 169.0), C-3 (δ 168.9) and correlations from H-1'' (δ 2.44) to C-4 (δ 101.7) confirmed that two alkyl chains were located at C-2 and C-5 of the α-pyrone moiety, respectively. The two side chains were assigned by the NOESY and HSQC-TOCSY spectrum, individually. Thus, compound **3** was determined to be pseudopyronine C[22].

Compound **4** was isolated as a white, amorphous powder and identified as labradorin 1 based on the NMR data, X-ray and HRESIMS data. Compound **5** was obtained as a white solid, and determined to be labradorin 2 by comparing the HRESIMS and NMR data with previous report[24], similarly, compound **6** was identified as pimprinaphine[25].

Figure S1 HRESIMS spectrum of known compound **1**

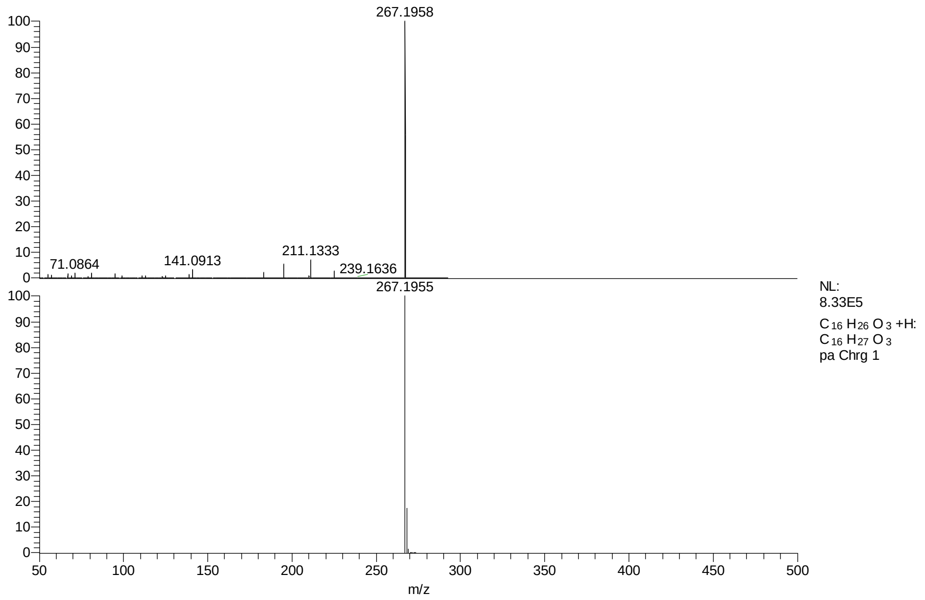

Figure S2 ^{1}H-NMR spectrum of known compound **1** (Methanol-*d*4, 600 MHz)

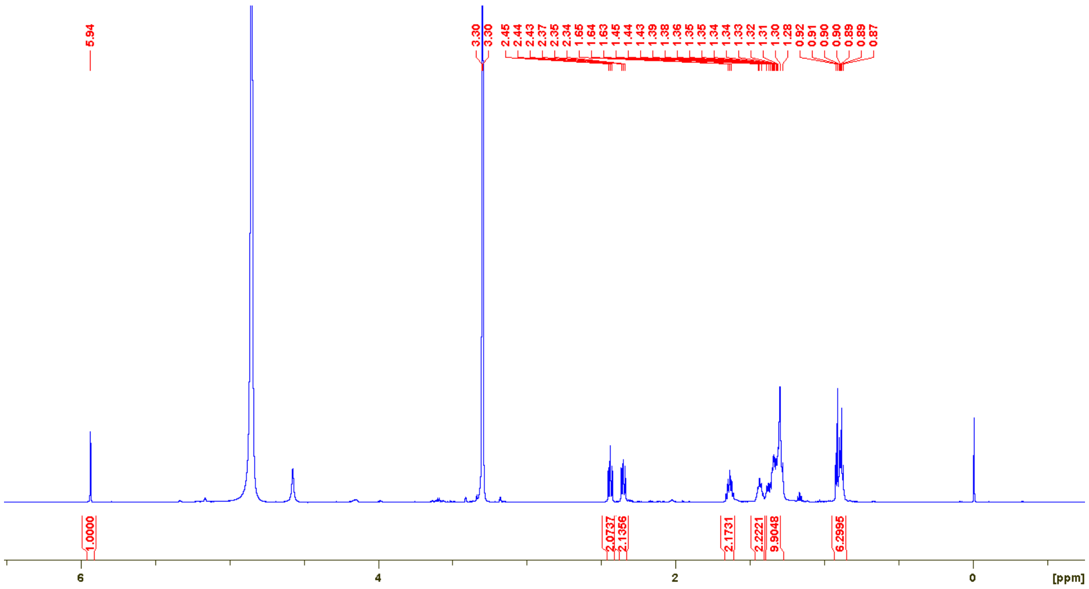

Figure S3 ^{13}C-NMR spectrum of known compound **1** (Methanol-d_4, 150 MHz)

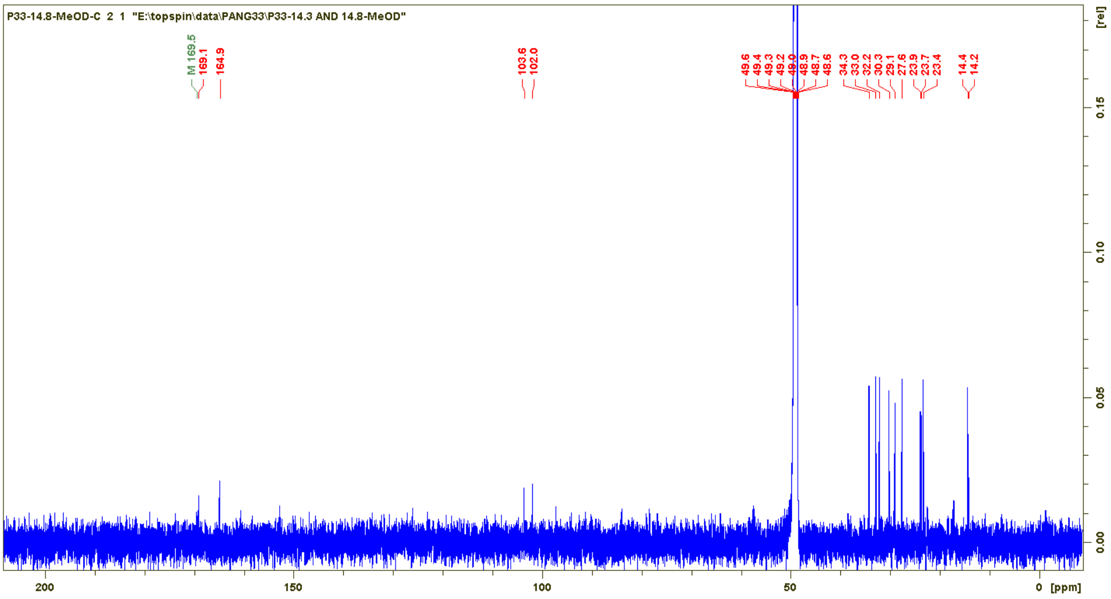

Figure S4 HRESIMS spectrum of known compound **2**

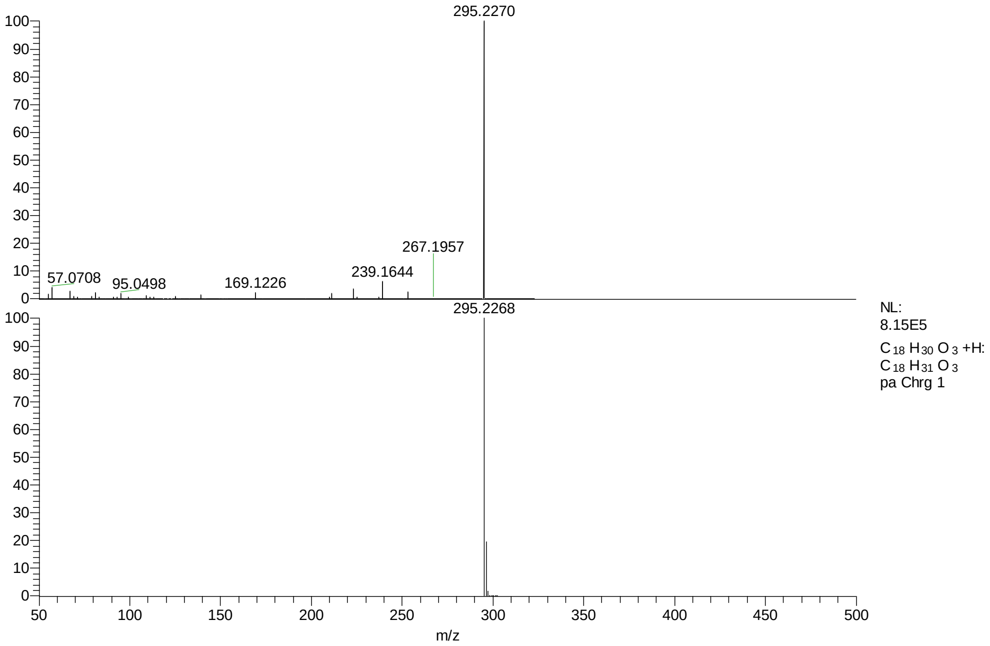

Figure S5 ^{1}H-NMR spectrum of known compound **2** ($CDCl_3$, 600 MHz)

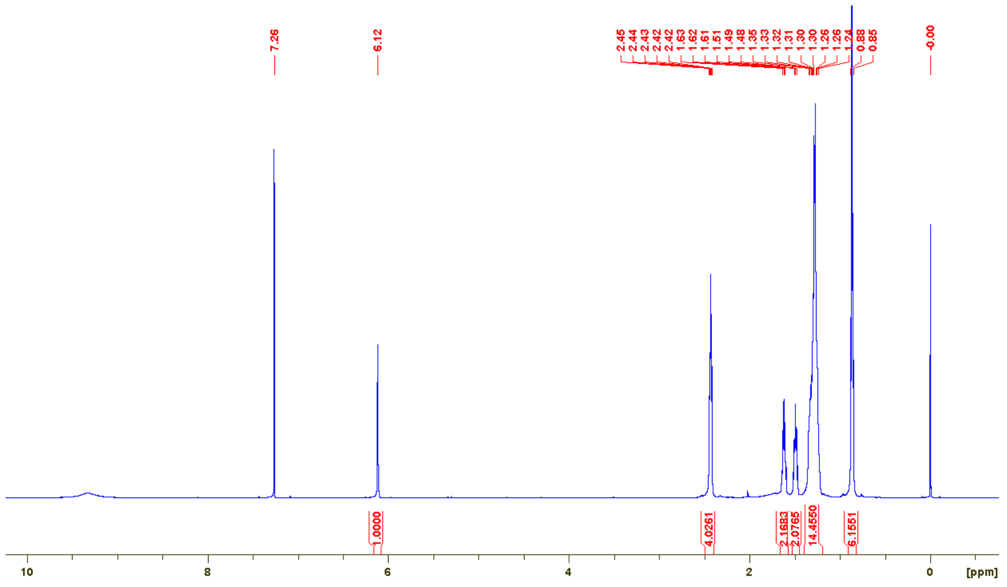

Figure S6 ^{13}C-NMR spectrum of known compound **2** ($CDCl_3$, 150 MHz)

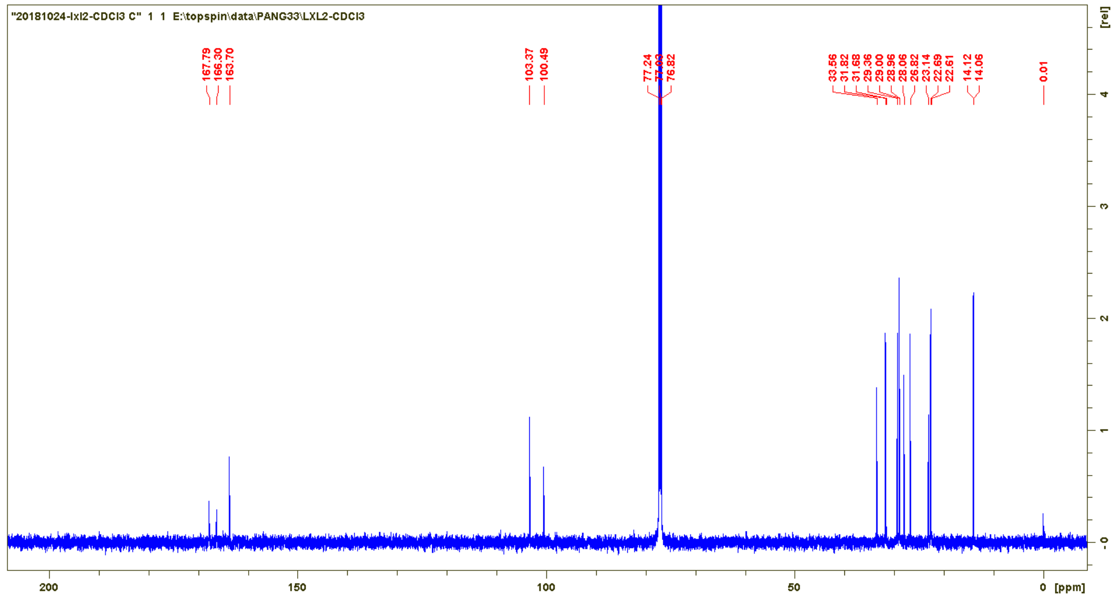

Figure S7 HRESIMS spectrum of known compound **3**

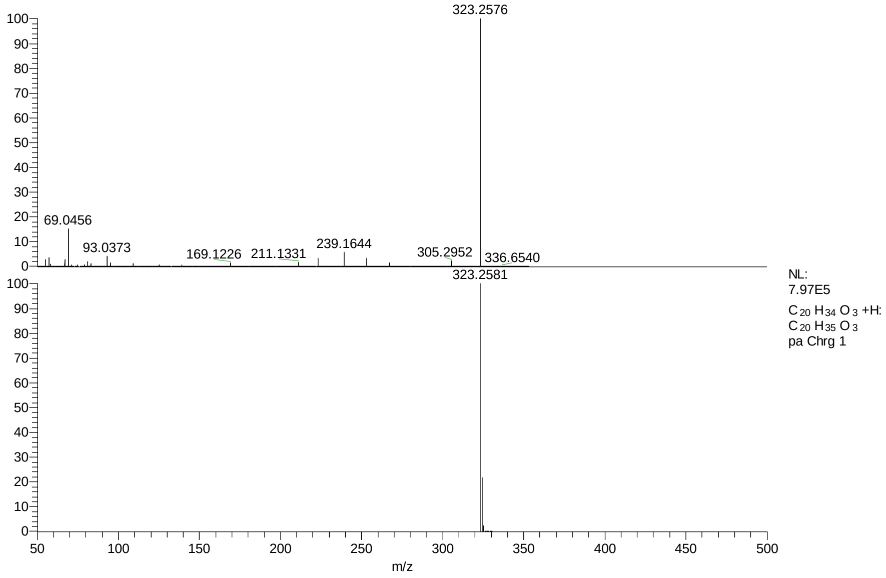

Figure S8 ^{1}H-NMR spectrum of known compound **3** (Methanol-*d*4, 600 MHz)

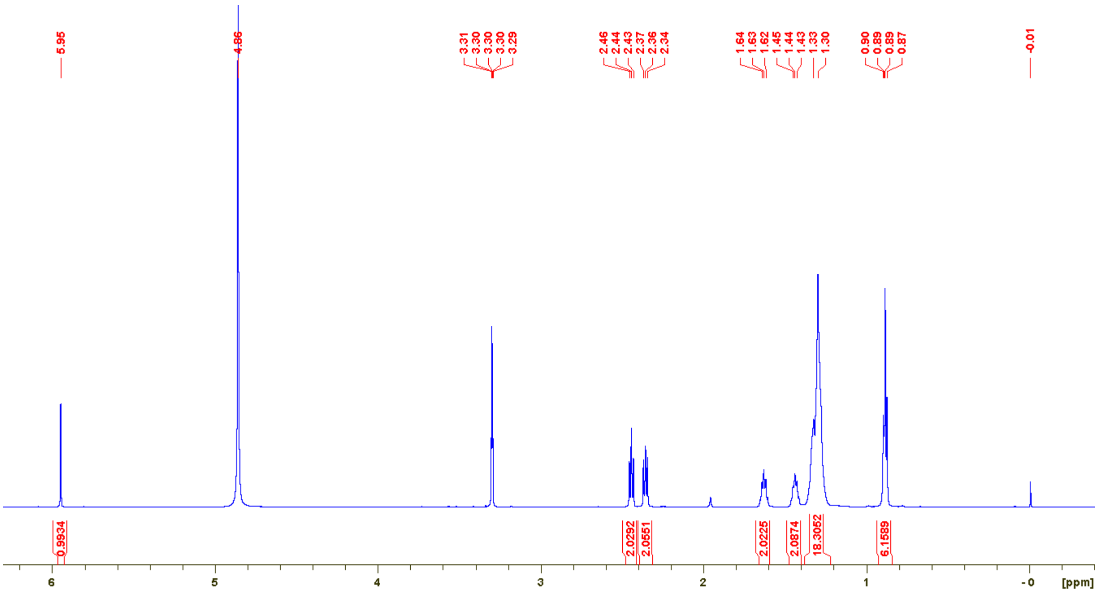

Figure S9 ^{13}C-NMR spectrum of known compound **3** (Methanol-d_4, 150 MHz)

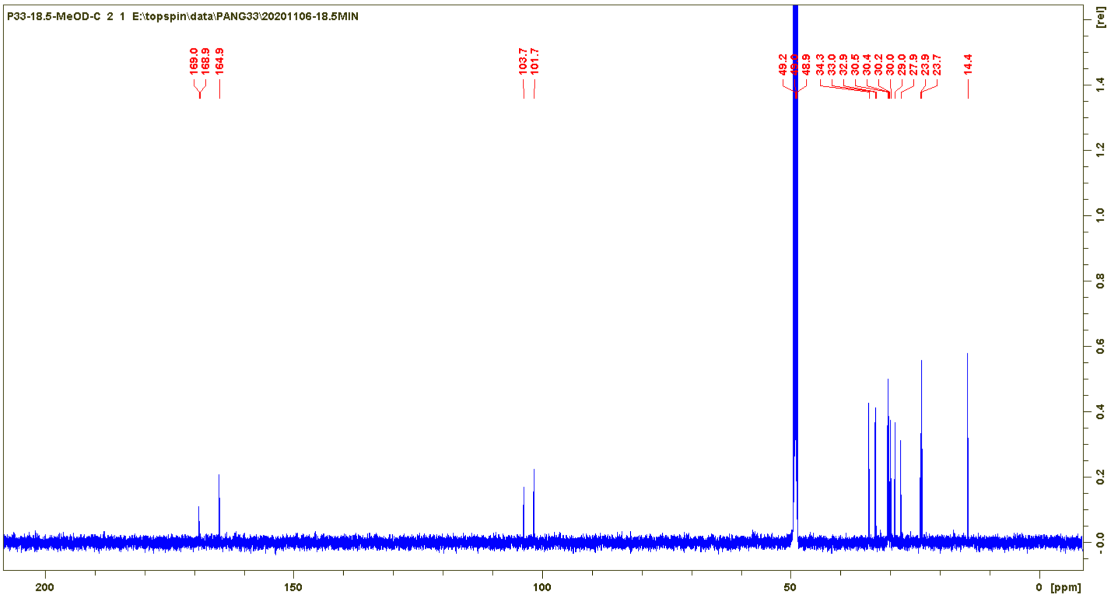

Figure S10 HRESIMS spectrum of known compound **4**

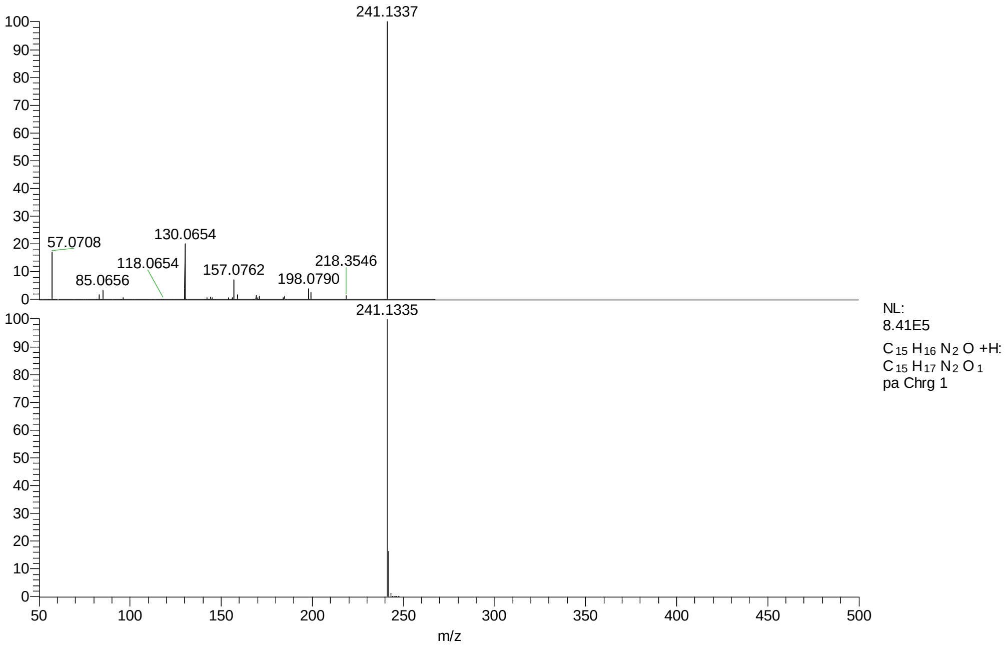

Figure S11 ^{1}H-NMR spectrum of known compound **4** (Methanol-d_4, 600 MHz)

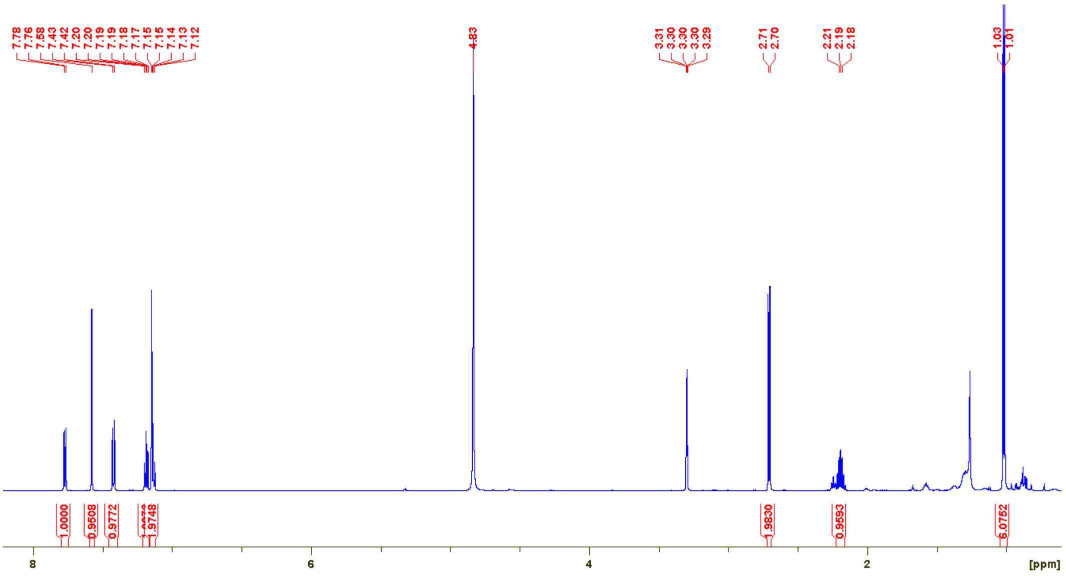

Figure S12 ^{13}C-NMR spectrum of known compound **4** (Methanol-d_4, 150 MHz)

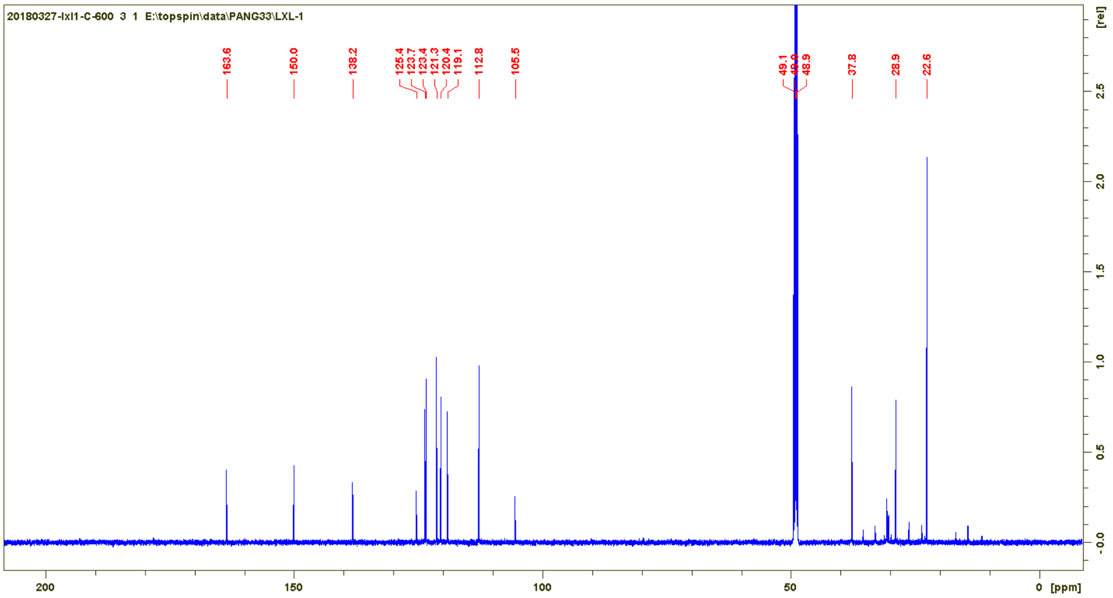

Figure S13 HRESIMS spectrum of known compound **5**

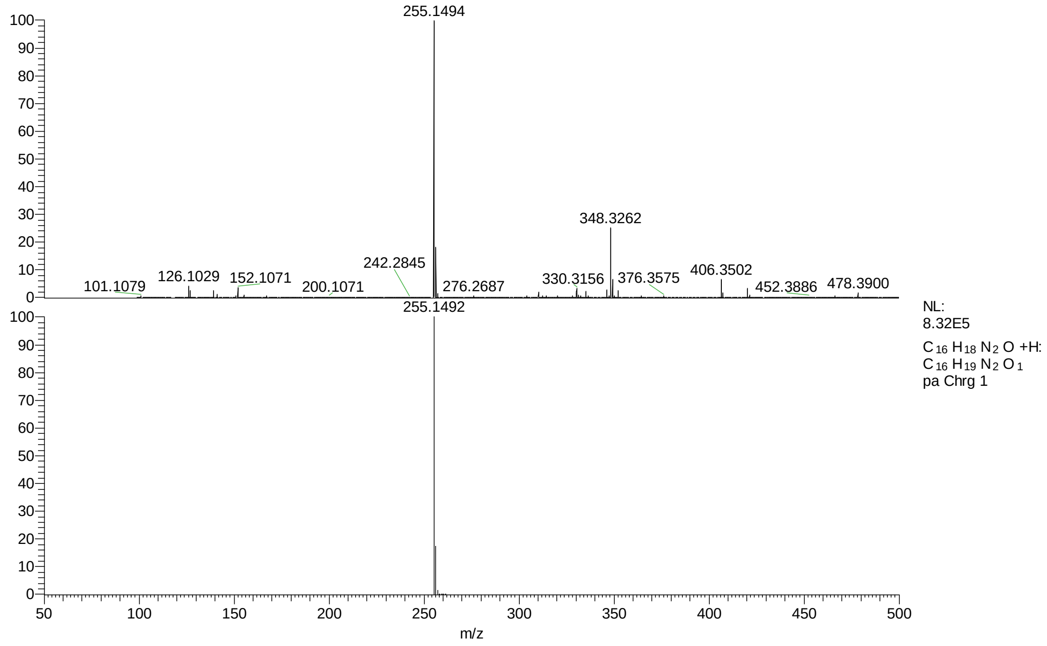

Figure S14 ^{1}H-NMR spectrum of known compound **5** (Methanol-d_4, 600 MHz)

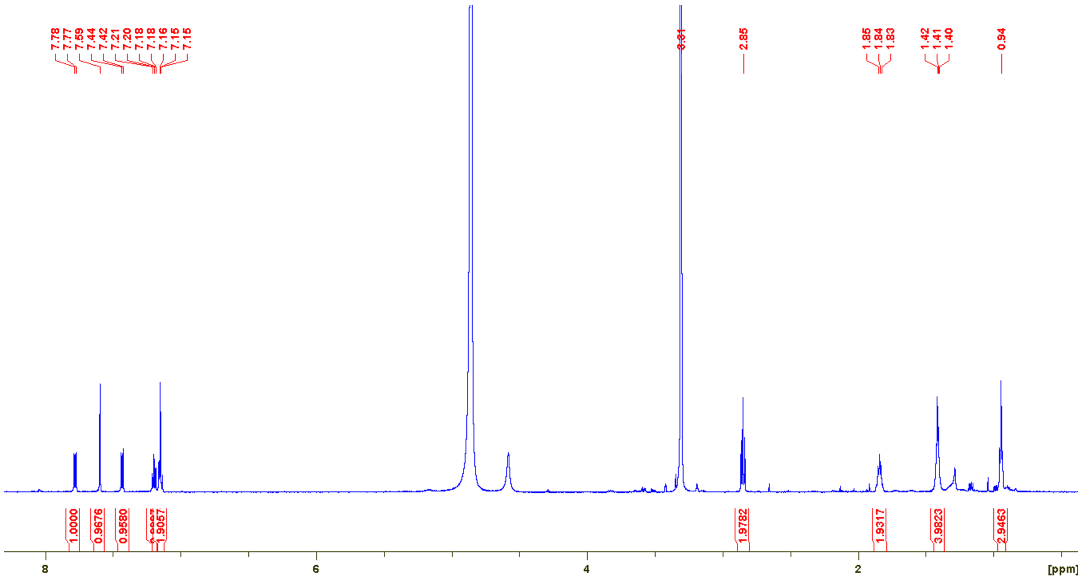

Figure S15 ^{13}C-NMR spectrum of known compound **5** (Methanol-d_4, 150 MHz)

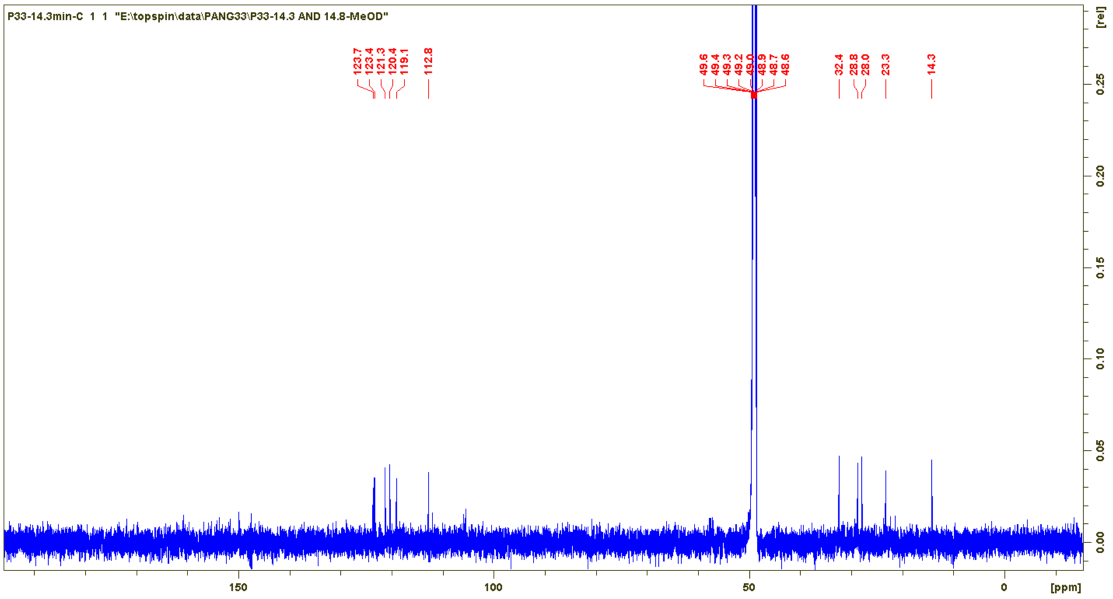

Figure S16 HRESIMS spectrum of known compound **6**

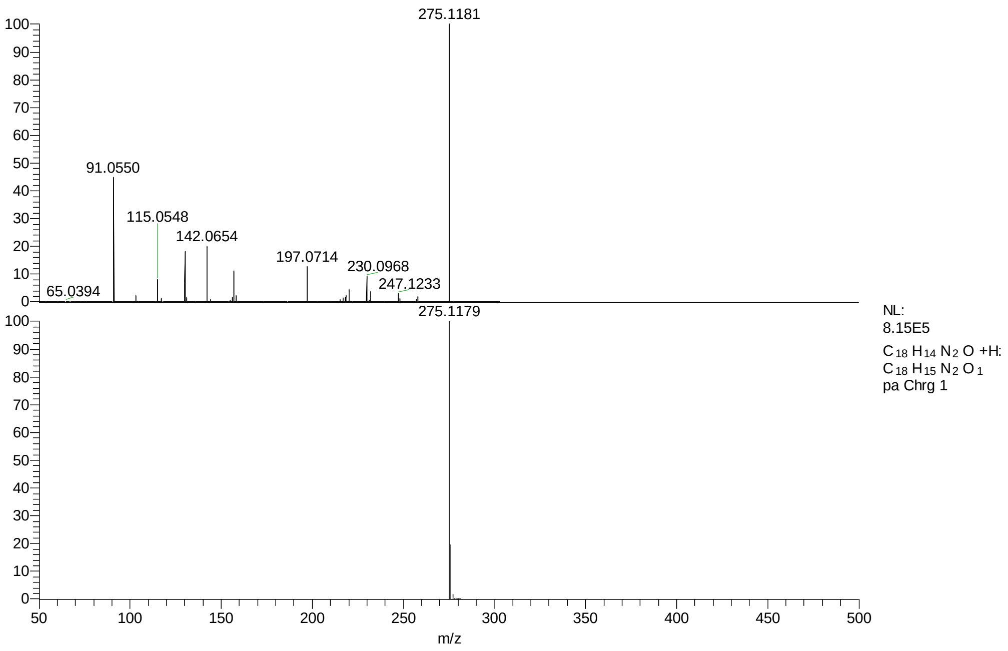

Figure S17 ^{1}H-NMR spectrum of known compound **6** ($CDCl_3$, 600 MHz)

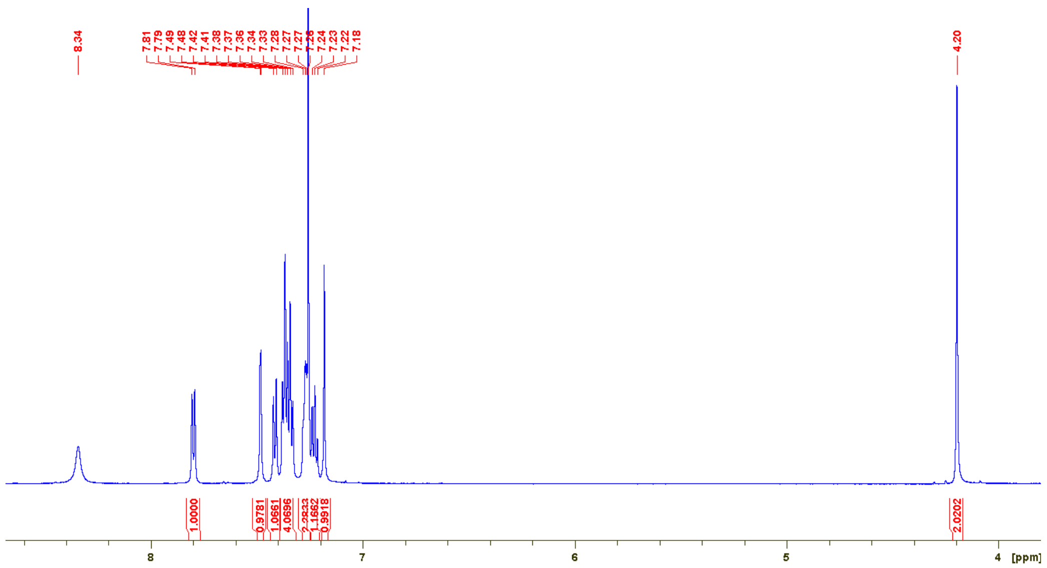

Figure S18 ^{13}C-NMR spectrum of known compound **6** ($CDCl_3$, 150 MHz)

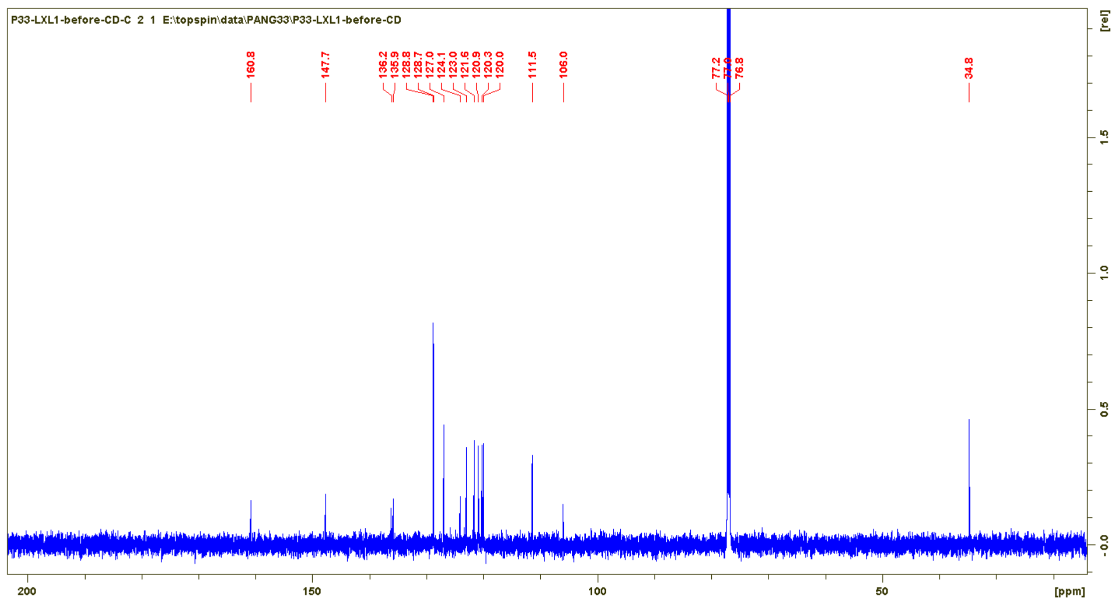

Figure S19 Antimicrobial assay of methanolic extract of *P. mosselii* P33

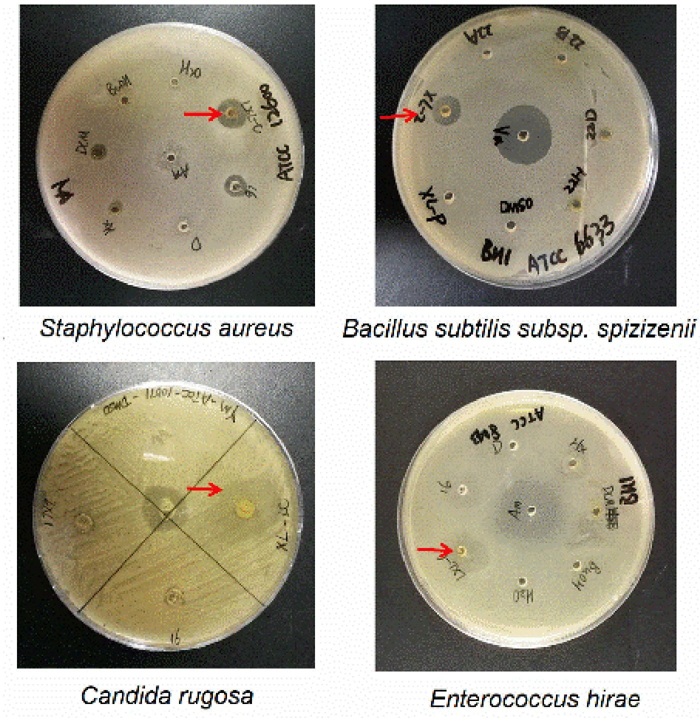

Figure S20 Phylogenetic tree of *P. mosselii* P33

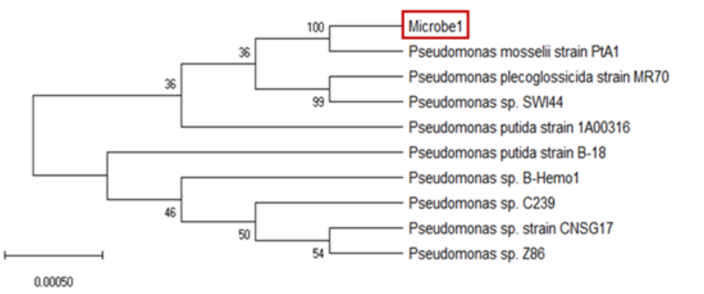

16S rDNA sequence of P33 was obtained as follows:

CCGTGGTAACCGTCCCCCCGAAGGTTAGACTAGCTACTTCTGGTGCAACCCACTCCCA
TGGTGTGACGGGCGGTGTGTACAAGGCCCGGGAACGTATTCACCGCAACATTCTGATT
TGCGATTACTAGCGATTCCGACTTCACGCAGTCGAGTTGCAGACTGCGATCCGGACTA
CGATCGGTTTTGTGAGATTAGCTCCACCTCGCGGCTTGGCAACCCTCTGTACCGACCAT
TGTAGCACGTGTGTAGCCCAGGCCGTAAGGGCCATGATGACTTGACGTCATCCCCACC
TTCCTCCGGTTTGTCACCGGCAGTCTCCTTAGAGTGCCCACCATAACGTGCTGGTAACT
AAGGACAAGGGTTGCGCTCGTTACGGGACTTAACCCAACATCTCACGACACGAGCTG
ACGACAGCCATGCAGCACCTGTGTCAGAGTTCCCGAAGGCACCAATCCATCTCTGGA
AAGTTCTCTGCATGTCAAGGCCTGGTAAGGTTCTTCGCGTTGCTTCGAATTAAACCACA
TGCTCCACCGCTTGTGCGGGCCCCCGTCAATTCATTTGAGTTTTAACCTTGCGGCCGTA
CTCCCCAGGCGGTCAACTTAATGCGTTAGCTGCGCCACTAAAATCTCAAGGATTCCAA
CGGCTAGTTGACATCGTTTACGGCGTGGACTACCAGGGTATCTAATCCTGTTTGCTCCC
CACGCTTTCGCACCTCAGTGTCAGTATCAGTCCAGGTGGTCGCCTTCGCCACTGGTGTT
CCTTCCTATATCTACGCATTTCACCGCTACACAGGAAATTCCACCACCCTCTACCATAC
TCTAGCTCGCCAGTTTTGGATGCAGTTCCCAGGTTGAGCCCGGGGCTTTCACATCCAA
CTTAACGAACCACCTACGCGCGCTTTACGCCCAGTAATTCCGATTAACGCTTGCACCCT
CTGTATTACCGCGGCTGCTGGCACAGAGTTAGCCGGTGCTTATTCTGTCGGTAACGTCA
AAACAGCAAGGTATTAGCTTACTGCCCTTCCTCCCAACTTAAAGTGCTTTACAATCCGA
AGACCTTCTTCACACACGCGGCATGGCTGGATCAGGCTTTCGCCCATTGTCCAATATTC
CCCACTGCTGCCTCCCGTAGGAGTCTGGACCGTGTCTCAGTTCCAGTGTGACTGATCAT
CCTCTCAGACCAGTTACGGATCGTCGCCTAGGTGAGCCATTACCTCACCTACTAGCTAA
TCCGACCTAGGCTCATCTGATAGCGCAAGGCCCGAAGGTCCCCTGCTTTCTCCCGTAG
GACGTATGCGGTATTAGCGTTCCTTTCGAAACGTTGTCCCCCACTACCAGGCAGATTCC
TAGGCATTACTCACCCGTCCGCCGCTGAATCAAGGAGCAA
